# Integrative Functional Genomics Implicated the Key T-/B-Cell Deficiency Regulator *RAG1* in Transarterial Chemoembolization of Hepatocellular Carcinoma

**DOI:** 10.3389/fcell.2021.720791

**Published:** 2021-09-27

**Authors:** Yeyang Xu, Teng Wang, Jiajia Zeng, Bowen Wang, Liqing Zhou, Ming Yang, Li Zhang, Nasha Zhang

**Affiliations:** ^1^Cheeloo College of Medicine, Shandong University, Jinan, China; ^2^Department of Radiation Oncology, Shandong Cancer Hospital and Institute, Shandong First Medical University and Shandong Academy of Medical Sciences, Jinan, China; ^3^Department of Radiation Oncology, Huaian No. 2 Hospital, Huaian, China; ^4^Shandong Provincial Key Laboratory of Radiation Oncology, Cancer Research Center, Shandong Cancer Hospital and Institute, Shandong First Medical University and Shandong Academy of Medical Sciences, Jinan, China; ^5^Department of Oncology, Tongji Hospital, Tongji Medical College, Huazhong University of Science and Technology, Wuhan, China

**Keywords:** RAG1, hepatocellular carcinoma, TACE, eQTL, TCGA, genetic polymorphism

## Abstract

Transarterial chemoembolization (TACE) has significantly prolonged overall survival (OS) of unresectable hepatocellular carcinoma (HCC) patients. Unfortunately, there are still a portion of patients without therapeutic responses to TACE. Although genome-wide association studies identified multiple HCC susceptibility SNPs, it is still largely unclear how genome-wide identified functional SNPs impacting gene expression contribute to the prognosis of TACE-treated HCC patients. In this study, we developed an integrative functional genomics methodology to identify gene expression-related SNPs significantly contributing to prognosis of TACE-treated HCC patients across the whole genome. Employing integration of data from expression quantitative trait locus (eQTLs) analyses of The Cancer Genome Atlas (TCGA) liver hepatocellular carcinoma (LIHC) as well as the 1000 Genomes project, we successfully annotated 60 gene expression-related SNPs which are associated with OS of the TCGA patients. After genotyping these 60 SNPs in our TACE cohort, we identified four SNPs (rs12574873, rs12513391, rs34597395, and rs35624901) which are significantly associated with OS of HCC patients treated with TACE. For instance, multivariate Cox proportional hazards model indicated that the rs35624901 Deletion.Deletion (Del.Del) genotype carriers had markedly prolonged OS and a 55% decreased death risk compared with individuals with the GG genotype after TACE therapy (*p* = 8.3 × 10^–5^). In support of this, the rs35624901 Del.Del genotype is correlated to higher expression of *RAG1*, a key T-/B-cell deficiency regulator. Our findings reported the first evidence supporting the prognostic value of four eQTL SNPs in TACE-treated HCC patients. Importantly, our data implicated that antitumor immunity might contribute to TACE efficiency for unresectable HCC patients.

## Introduction

Hepatocellular carcinoma (HCC) accounts for ninety percent of primary liver tumors and is one of the deadliest malignancies worldwide, especially in Asia (Llovet et al., 2016; Ferlay et al., 2019). Hepatitis due to infection from hepatitis B virus and/or hepatitis C virus, intakes of aflatoxin B1, heavy alcohol drinking, and smoking has been reported to be well-established risk factors of HCC (Llovet et al., 2016; Craig et al., 2019). Most HCC patients are commonly diagnosed at advanced stages and have poor overall survival (OS; Llovet et al., 2016; Ferlay et al., 2019). For these unresectable HCC patients, transarterial chemoembolization (TACE) has been widely used in clinic. TACE promotes ischemic necrosis of tumors by blocking the arterial supply of the tumor and simultaneously delivering a cytotoxic chemotherapeutic agent. The key randomized controlled clinical trials demonstrated that TACE improved OS of unresectable HCC patients (Llovet et al., 2002; Lo et al., 2002). For instance, in Asian patients with unresectable HCC, transarterial Lipiodol chemoembolization is an effective treatment form and could significantly prolong OS (Lo et al., 2002), while not all unresectable HCC patients could benefit from TACE treatment and some even showed rapid disease progression after TACE (Llovet et al., 2002; Lo et al., 2002). Therefore, identification of novel biomarkers for patient-tailored TACE is urgently needed.

Single nucleotide polymorphisms (SNPs) are the most common type of genetic variants in human. Genome-wide association studies (GWAS) identified multiple SNPs associated with HCC susceptibility and prognosis (Zhang et al., 2010; Kumar et al., 2011; Miki et al., 2011; Li et al., 2012). Interestingly, most GWAS-identified HCC-risk or HCC-prognostic SNPs might function *via* regulating gene expression due to their location in non-coding regions of human genome (Hindorff et al., 2009). As a result, interpreting mechanisms of SNP-regulated gene expression is crucial for understanding the biological nature of these HCC-related SNPs (Gong et al., 2018). As a powerful tool to disclose detailed impacts of functional SNPs in cancers, expression quantitative trait locus (eQTL) analysis links SNP genotypes to gene expression in various tissues (Grundberg et al., 2012; Westra et al., 2013; Zhu et al., 2016). Although candidate gene studies declared the value of SNPs as prognostic biomarkers for HCC treated with TACE (Zhao et al., 2012; Tang et al., 2014; Yu et al., 2014; Yuan et al., 2014; Wu et al., 2015; Liu et al., 2016; Qiu and Liu, 2016), it is still unclear how genome-wide identified functional SNPs impacting gene expression contribute to prognosis of HCC patients undergoing TACE. Therefore, we systematically screened gene expression-related genetic variants *via* survival-associated eQTLs in The Cancer Genome Atlas (TCGA) liver hepatocellular carcinoma (LIHC) patients. There are 825 candidate SNPs with minor allele frequency (MAF) ≥5% in Han Chinese populations according to the 1,000 Genomes project. We then genotyped 60 candidate genetic variants in the Jiangsu HCC TACE cohort and identified four SNPs which significantly contributed to the prognosis of HCC patients after TACE in Chinese Han population.

## Materials and Methods

### SNP Selection

The gene expression-related genetic variants associated with OS of LIHC patients in TCGA were identified using the PancanQTL database^1^. Both *cis*-eQTLs and *trans*-eQTLs in HCC tissues were included in the current study. SNPs with MAF less than 0.05 in Han Chinese populations were excluded using information from the 1,000 Genomes project database.

### Study Subjects

This study consisted of 273 Stage III or IV HCC Han Chinese patients treated with TACE. The detailed characteristics of all patients have been reported previously (Liu et al., 2021). All HCC patients were recruited at Huaian No. 2 Hospital (Huaian, Jiangsu Province, China) between January 1999 and January 2013. All patients had histologically confirmed HCC with clinical data available. All subjects had no history of other cancers. Within a week after diagnosis, all patients underwent TACE as the first-line therapy. For most HCC cases, both oxaliplatin and doxorubicin were used for TACE. OS was calculated from the date of diagnosis to the date of death for any cause. At recruitment, written informed consent was obtained from each patient. This study was approved by the Institutional Review Board of Huaian No.2 Hospital. The methods were carried out in accordance with the approved guidelines.

### Genotyping

A total of 60 candidate SNPs were genotyped in Jiangsu TACE cohort and 58 candidate SNPs were successfully genotyped (Table 1). Genotypes of these fifty-eight polymorphisms were determined using the iPLEX Sequenom MassARRAY platform (Sequenom) as previously reported (Li Y. et al., 2020; Li Z. et al., 2020). A 15% random sample was reciprocally tested and the reproducibility was 100%.

**TABLE 1:** Log-rank and Cox-regression analyses of 60 PancanQTL-identified candidate genetic variants for OS in the Jiangsu cohort.

No	rsID	Log-rank p-Value	HR	95% CI	Cox *p*-value
1	rs1001530	0.823	0.939	0.75–1.17	0.576
2	rs111537617	0.654	0.902	0.72–1.13	0.375
3	rs113791471	0.822	1.066	0.86–1.32	0.555
4	rs116064696	0.905	0.941	0.71–1.25	0.675
5	rs11644804	0.106	0.917	0.74–1.14	0.426
6	rs11711287	0.809	1.029	0.85–1.24	0.77
7	rs1179087	0.64	0.896	0.70–1.15	0.386
8	rs12513391	2.3 × 10^–6^	1.61	1.30–1.99	1.0 × 10^–5^
9	rs12551668	0.71	1.01	0.80–1.28	0.928
10	rs12793220	0.093	1.04	0.81–1.33	0.778
11	rs13306519	0.204	0.83	0.68–1.03	0.088
12	rs141915154	0.925	0.97	0.81–1.15	0.709
13	rs147341020	0.002	1.11	0.86–1.43	0.421
14	rs16929939	0.161	0.82	0.66–1.01	0.063
15	rs17097827	0.137	1.25	0.99–1.57	0.057
16	rs17837965	0.777	1.1	0.83–1.46	0.506
17	rs2163539	0.708	1.07	0.90–1.27	0.429
18	rs35624901	2.8 × 10^–5^	0.61	0.48–0.76	1.9 × 10^–5^
19	rs4128191	0.465	0.94	0.80–1.11	0.483
20	rs4644835	0.467	0.89	0.66–1.22	0.477
21	rs4784063	0.777	1.08	0.87–1.35	0.488
22	rs4823082	0.291	0.85	0.65–1.11	0.226
23	rs61662147	0.064	0.84	0.69–1.03	0.089
24	rs7080745	0.533	1.15	0.89–1.48	0.69
25	rs73214543	0.135	0.96	0.78–1.17	0.658
26	rs73219251	genotyping failed			
27	rs7620445	0.476	1.12	0.93–1.34	0.243
28	rs76241969	genotyping failed			
29	rs9550238	0.218	1.01	0.81–1.26	0.911
30	rs9624873	0.931	1.03	0.79–1.34	0.825
31	rs10189492	0.508	0.99	0.79–1.23	0.905
32	rs10786361	0.24	0.81	0.61–1.07	0.129
33	rs10971392	0.386	1.14	0.92–1.40	0.233
34	rs11168866	0.264	0.97	0.82–1.14	0.723
35	rs11253104	0.573	1.09	0.91–1.29	0.361
36	rs11281227	0.947	1	0.84–1.20	0.965
37	rs11620307	0.429	0.9	0.73–1.10	0.302
38	rs11642999	0.001	0.92	0.69–1.21	0.546
39	rs12552290	0.628	1.08	0.87–1.33	0.479
40	rs12574873	0.018	1.39	1.11–1.75	0.005
41	rs13146617	0.844	1	0.76–1.32	0.985
42	rs1394802	0.261	1.01	0.82–1.23	0.936
43	rs1463093	0.366	1.14	0.92–1.42	0.228
44	rs148073299	0.07	1.17	0.97–1.42	0.103
45	rs156112	0.139	1.23	0.99–1.52	0.056
46	rs16920343	0.782	0.94	0.71–1.25	0.675
47	rs199498726	0.34	1.03	0.84–1.26	0.809
48	rs2005618	0.594	0.95	0.80–1.12	0.539
49	rs226492	0.563	0.89	0.71–1.11	0.301
50	rs2288341	0.198	0.95	0.72–1.24	0.699
51	rs2385911	0.07	1	0.78–1.29	0.973
52	rs272135	0.403	0.88	0.72–1.08	0.21
53	rs2849605	0.691	1.02	0.77–1.35	0.9
54	rs34597395	0.005	1.25	1.04–1.50	0.019
55	rs3826405	0.896	0.94	0.72–1.24	0.67
56	rs6464773	0.667	1.05	0.85–1.30	0.638
57	rs6597827	0.908	0.98	0.72–1.33	0.91
58	rs74049184	0.545	0.99	0.79–1.23	0.895
59	rs74567609	0.788	1.09	0.83–1.43	0.543
60	rs9838251	0.952	0.97	0.80–1.18	0.784

*eQTLs, expression quantitative trait loci; OS, overall survival time; HR, hazard ratio; CI, confidence interval.*

### Statistics

Survival curves were compared between different genotypes using the log-rank test. The impacts of genotypes on OS were examined using the Kaplan–Meier method. Multivariate prognostic factors for OS were analyzed using Cox regression analyses. Hazard ratios (HRs) and 95% confidence intervals (CIs) for the association between the candidate SNPs and death risk of HCC patients were adjusted for age of onset, sex, smoking status, drinking status, hepatitis history, stage, and HCC family history, where they were appropriate. A *p*-value of less than 0.05 was used as the criterion of statistical significance. All statistical tests were two-sided. All analyses were performed with GraphPad or SPSS software package.

## Results

### Genome-Wide Identification of eQTLs SNPs Associated With Survival of the TCGA LIHC Patients

**FIGURE 1:**
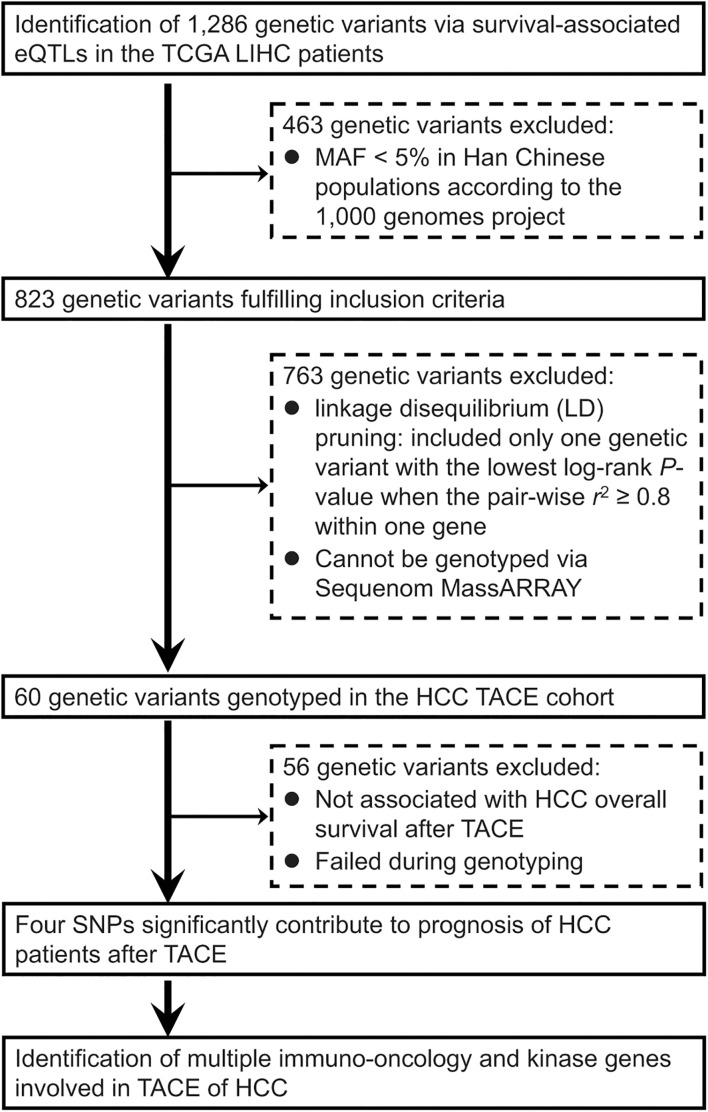
Flowchart of an integrative functional genomics methodology to identify survival-related expression quantitative trait locus (eQTL) genetic variants involved in the transarterial chemoembolization (TACE) treatment of hepatocellular carcinoma (HCC) across the whole genome.

As summarized in Figure 1, we conducted a genome-wide screening of gene expression-related genetic polymorphisms (eQTLs SNPs) which are significantly associated with OS of the TCGA LIHC patients. First, a total of 1,286 LIHC survival-associated eQTLs SNPs were identified using the PancanQTL database (all log-rank *p* < 4.9 × 10^–4^; all eQTLs *p* < 1.7 × 10^–4^). Next, according to the 1,000 Genomes project, 463 genetic variants were excluded due to their MAFs less than 5% in Han Chinese populations. A total of 763 genetic polymorphisms were then excluded based on linkage disequilibrium (LD) pruning and genotyping feasibility *via* Sequenom MassARRAY. For LD pruning, we only included one genetic variant with the lowest log-rank *p*-value when the pairwise *r*^2^ ≥ 0.8 within one gene. Eight SNPs could not be genotyped *via* Sequenom MassARRAY due to the presence of highly homologous DNA sequences in human genome. In particular, one of the important steps during Sequenom MassARRAY is to amplify a DNA fragment containing the candidate SNP using PCR. However, specific primers cannot be designed for PCR due to highly homologous DNA sequences around the SNP that exist in other gene loci of the human genome. This screening finally resulted in a total of 60 eQTLs SNPs associated with OS of the TCGA LIHC patients (Table 1).

### Characteristics of HCC Patient Undergoing TACE

The median age of HCC patients treated with TACE was 56 years (range, 25–81 years). There were 232 males (85.0%), 75 smokers (27.5%), 77 drinkers, 169 individuals with hepatitis history (61.9%), and 18 patients with HCC family history (6.6%). Among these patients, 72 (26.4%) had stage III diseases and 201 (73.6%) had stage IV diseases. At the final analysis, all patients died and the median OS time was 20 months (range, 1–105 months).

### Effects of eQTLs Genetic Polymorphisms on OS of TACE-Treated HCC Patients

**FIGURE 2:**
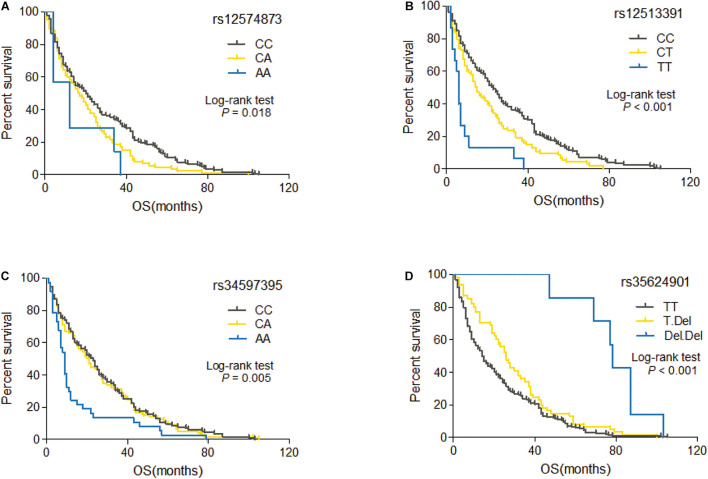
Kaplan–Meier curve of OS for TACE-treated HCC patients with different genotypes of rs12574873 **(A)**, rs12513391 **(B)**, rs34597395 **(C)**, or rs35624901 **(D)**.

Among 60 candidate SNPs, only four genetic variants (rs12574873, rs12513391, rs34597395, and rs35624901) are significantly associated with OS of HCC patients treated with TACE (rs12574873: log-rank *p* = 0.018, unadjusted Cox *p* = 0.005; rs12513391: log-rank *p* = 2.3 × 10^–6^, unadjusted Cox *p* = 1.0 × 10^–5^; rs34597395: log-rank *p* = 0.005, unadjusted Cox *p* = 0.019; rs35624901: log-rank *p* = 2.8 × 10^–5^, unadjusted Cox *p* = 1.9 × 10^–5^) (Table 2). As shown in Figure 2A, TACE-treated HCC patients with the rs12574873 CA or AA genotype had a lower survival than the cases with the CC genotype (log-rank *p* = 0.018). Similarly, the HCC cases with the rs12513391 CT or TT genotype had a shorter survival time than the patients carrying the CC genotype (log-rank *p* < 0.001) after TACE treatment (Figure 2B). Although there were no obvious differences for OS time between rs34597395 CC and CA genotypes, Kaplan–Meier survival estimates indicated that the rs34597395 AA genotype carriers had a lower survival than the HCC cases carrying the CC or CA genotype after TACE (log-rank *p* = 0.005) (Figure 2C). Moreover, TACE-treated HCC patients with the rs35624901 Deletion.Deletion (Del.Del) genotype had markedly prolonged survival time than the individuals carrying the TT or T.Del genotype (log-rank *p* < 0.001) (Figure 2D).

**TABLE 2:** Multivariate Cox-regression analyses of PancanQTL-identified genetic variants for OS in the Jiangsu cohort.

SNP IDs	Genotypes	Patients	Jiangsu cohort	
		*n* (%)	HR (95% CI)	*p*-Value
	CC	179 (65.6)	Reference	
rs12574873	CA	87 (31.8)	1.33 (1.01–1.74)	0.044
	AA	7 (2.6)	1.32 (0.89–1.95)	0.169
	CC	165 (60.4)	Reference	
rs12513391	CT	93 (34.1)	1.46 (1.12–1.90)	0.005
	TT	15 (5.5)	1.80 (1.35–2.39)	5.7 × 10^–5^
	CC	115 (42.1)	Reference	
rs34597395	CA	121 (44.3)	1.05 (0.80–1.37)	0.731
	AA	37 (13.6)	1.43 (1.18–1.74)	3.1 × 10^–4^
	TT	205 (75.1)	Reference	
rs35624901	T.Del	61 (22.3)	0.75 (0.56–0.99)	0.047
	Del.Del	7 (2.6)	0.45 (0.30–0.67)	8.3 × 10^–5^

*SNP, single nucleotide polymorphism; OS, overall survival time; HR, hazard ratio; CI, confidence interval; Del, deletion.*

*^∗^HRs and 95% CIs for the association between clinical variables and death risk was adjusted for age of onset, sex, smoking status, drinking status, hepatitis history, stage, and HCC family history, where it was appropriate.*

Multivariate Cox proportional hazards model showed that the rs12574873, rs12513391, and rs34597395 SNPs were significantly associated with increased death risk of TACE-treated HCC patients, and rs35624901 markedly contributed to decreased death risk (Table 2). Compared with the CC genotype, the rs12574873 CA genotype was significantly associated with elevated death risk (HR = 1.33, 95% CI = 1.01–1.74, *p* = 0.044). The rs12513391 CT or TT genotype was significantly associated with increased death risk after TACE treatment compared with the CC genotype (the CT genotype: HR = 1.46, 95% CI = 1.12–1.90, *p* = 0.005; the TT genotype: HR = 1.80, 95% CI = 1.35–2.39, *p* = 5.7 × 10^–5^). Similarly, TACE-treated HCC patients with the rs34597395 AA genotype had a 43% increased death risk compared with patients carrying the CC genotype (95% CI = 1.18–1.74, *p* = 3.1 × 10^–4^). On the contrary, the rs35624901 T.Del or Del.Del genotype carriers had a 25% or 55% decreased death risk compared with individuals with the GG genotype after TACE therapy (95% CI = 0.56–0.99, *p* = 0.047; 95% CI = 0.30–0.67, *p* = 8.3 × 10^–5^).

### Impacts of Four eQTLs SNPs on Gene Expression and OS of TCGA LIHC Patients

**FIGURE 3:**
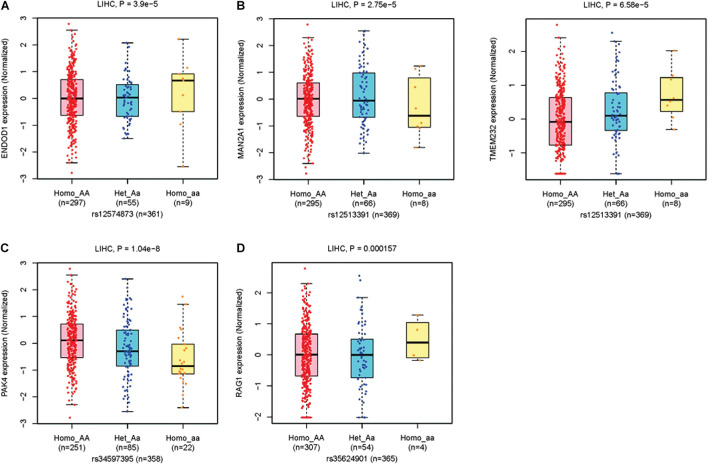
The boxplots of TCGA LIHC eQTLs. **(A)** The *ENDOD1* expression levels grouped by different genotypes of rs12574873. **(B)** The *MAN2A1* (left panel) and *TMEM232* (right panel) expression levels grouped by different genotypes of rs12513391. **(C)** The *PAK4* expression levels grouped by different genotypes of rs34597395. **(D)** The *RAG1* expression levels grouped by different genotypes of rs35624901.

For eQTLs analyses, the rs12574873 AA genotype is evidently associated with increased *ENDOD1* gene expression in TCGA LIHC patients compared to the CC or CA genotype (*p* = 3.9 × 10^–5^) (Figure 3A and Table 3). Interestingly, in these patients, the rs12513391 TT genotype is associated with both decreased *MAN2A1* gene expression and elevated *TMEM232* gene expression compared to the TT or CT genotype (for *MAN2A1*: *p* = 2.8 × 10^–5^; for *TMEM232*: *p* = 6.6 × 10^–5^) (Figure 3B and Table 3). Moreover, compared to the CC or CA genotype, the rs34597395 AA genotype is significantly associated with decreased *PAK4* gene expression in LIHC patients (*p* = 1.0 × 10^–8^) (Figure 3C and Table 3). On the contrary, there is obviously increased *RAG1* gene expression in the rs35624901 Del.Del genotype carriers compared with that in individuals with the TT or T.Del genotype (*p* = 1.6 × 10^–4^) (Figure 3D and Table 3).

**TABLE 3:** PancanQTL-identified genetic variants significantly associated with OS of TCGA LIHC patients.

#	SNP IDs	SNP position (hg19)	*n*	Genotypes	Gene symbol of eQTLs	*Cis*- or *Trans*-eQTLs	*p*-Value of eQTLs	MST (months)	Log-rank *p*-value
				CC				13.35	
1	rs12574873	chr11:95006396	190	CA	*ENDOD1*	*Cis*-eQTLs	3.9 × 10^–5^	19.5	3.6 × 10^–4^
				AA				3.03	
				CC				13.97	
2	rs12513391	chr5:109774372	192	CT	*MAN2A1*/*TMEM232*	*Cis*-eQTLs	2.8 × 10^–5/^ 6.6 × 10^–5^	8.67	1.0 × 10^–5^
				TT				0.65	
				CC				13.57	
3	rs34597395	chr21:15636780	189	CA	*PAK4*	*Trans*-eQTLs	1.0 × 10^–8^	14.43	3.7 × 10^–4^
				AA				7.43	
				TT				13.85	
4	rs35624901	chr11:35603111	190	T.Del	*RAG1*	*Cis*-eQTLs	1.6 × 10^–4^	1.83	9.7 × 10^–6^
				Del.Del				0	

*OS, overall survival; TCGA, The Cancer Genome Atlas; LIHC, liver hepatocellular carcinoma; SNP, single nucleotide polymorphism; MST, median overall survival time; eQTLs, expression quantitative trait loci; Del, deletion.*

Evident associations between these four genetic variants and OS of TCGA LIHC patients are also observed (Table 3). The median overall survival time (MST) of LIHC patients carrying the rs12574873 CC, CA, or AA genotype is 13.35, 19.5, or 3.03 months (log-rank *p* = 3.6 × 10^–4^). Similarly, MST of individuals with the rs12513391CC, CT, or TT genotype is 13.97, 8.67, or 0.65 months (log-rank *p* = 1.0 × 10^–5^). There is also markedly short OS time for HCC cases with the rs34597395 AA genotype compared to patients with the CC or CA genotype (MST AA vs. CC: 7.43 vs. 13.57 months; MST AA vs. CA: 7.43 vs. 14.43 months). Additionally, the TCGA LIHC patients carrying the T.Del genotype have a lower survival than the cases with the TT genotype (log-rank *p* = 9.7 × 10^–6^).

## Discussion

Hepatocellular carcinoma is a common kind of malignancy worldwide, especially in Asia. It remains difficult to cure HCC since patients were frequently diagnosed at advanced tumor stages (Llovet et al., 2016; Ferlay et al., 2019). Currently, there is no single treatment applicable to all HCC cases. TACE has been proven to be an effective palliative treatment for unresectable HCC (Llovet et al., 2002; Lo et al., 2002). In Asian patients, TACE resulted in a marked tumor response, and the 1-year survival was significantly better in the TACE group than in the control group (57 vs. 32%) (Lo et al., 2002). However, there were still 43% unresectable HCC patients who had progressive disease during TACE treatment in the first year (Lo et al., 2002). This suggests that further studies are required to identify biomarkers for patient-tailored therapy targeting individual genetic background correlated to the prognosis of HCC patients who received TACE.

Through a genome-wide eQTL strategy, we successfully identified four gene expression-related genetic variants which are significantly associated with OS of unresectable HCC patients treated with TACE. For instance, the rs35624901 Del.Del genotype is correlated to higher *RAG1* expression levels as well as prolonged OS of TACE-treated HCC patients. RAG1 is critical to initiate the V(D)J recombination which ensures appropriate assembly of antigen receptor genes in the correct cell lineage and in the proper developmental order (Schatz and Ji, 2011). Loss-of-function of *RAG1* due to nonsense mutations or gene knock-out abrogates T- and B-lymphocyte receptor formation prevents the maturation of T- and B-lymphocytes and leads to severe combined immunodeficiency (SCID; Schatz and Ji, 2011). *RAG1* plays a critical role in HCC development. Upon treatment of the chemical carcinogen diethylnitrosamine (DEN), progression of hepatic tumors was strikingly enhanced in T-/B cell-deficient *Rag1*(-/-) mice (Schneider et al., 2012). *Rag1*(-/-) mice showed markedly enhanced growth in the number and mass of hepatic tumors (Schneider et al., 2012). Consistently, we observed that TACE-treated HCC patients with low *RAG1* expression due to carrying the TT or T.Del genotype showed shorter OS compared to the Del.Del genotype carriers. This is biologically plausible. It has been reported that TACE did not significantly modify numbers of tumor-infiltrating lymphocytes (TILs) in HCC tissues as compared with the untreated condition in patients who received surgery (Craciun et al., 2020). Therefore, TACE-treated HCC patients with low *RAG1* expression may show low antitumor immune responses, rapid tumor progression, and thus, short OS time.

We enrolled 232 males (85.0%) in the current HCC TACE cohort. Consistently, we observed more male HCC patients in the two most famous randomized controlled trials on the role of TACE in HCC treatment (Llovet et al., 2002; Lo et al., 2002). In the study, for the first time, it was indicated that TACE improved the survival of stringently selected patients with unresectable HCC; there were 30 males (81%) in the Embolization group, 32 males (80%) in the Chemoembolization group, and 23 males (66%) in the Control group (Llovet et al., 2002). Similarly, in the Asian randomized controlled trial, there were 36 males (90%) in the Chemoembolization group and 34 males (87%) in the Control group (Lo et al., 2002).

In summary, our results reported the first evidence supporting the prognostic value of four eQTL genetic polymorphisms in HCC patients who received TACE therapy. Importantly, our integrative functional genomics indicated that T-/B-cell development-related *RAG1* may impact TACE efficiency *via* regulating antitumor immunity in HCC, and *RAG1* may be a target to improve therapeutic strategy for unresectable HCC patients.

## Data Availability Statement

The datasets presented in this study can be found in online repositories. The names of the repository/repositories and accession number(s) can be found in the article/supplementary material.

## Ethics Statement

The studies involving human participants were reviewed and approved by this study was approved by the Institutional Review Board of Huaian No. 2 Hospital. The patients/participants provided their written informed consent to participate in this study.

## Author Contributions

NZ designed the study. YX performed the experiments. NZ, YX, and LZha performed data analyses. MY, LZho, BW, TW, and JZ were responsible for the collection of specimens. NZ and LZha supervised the project. NZ and YX contributed to drafting the manuscript. All authors have read and approved the final submitted manuscript.

## Conflict of Interest

The authors declare that the research was conducted in the absence of any commercial or financial relationships that could be construed as a potential conflict of interest.

## Publisher’s Note

All claims expressed in this article are solely those of the authors and do not necessarily represent those of their affiliated organizations, or those of the publisher, the editors and the reviewers. Any product that may be evaluated in this article, or claim that may be made by its manufacturer, is not guaranteed or endorsed by the publisher.

## Footnotes

1
http://bioinfo.life.hust.edu.cn/PancanQTL/
